# An In Vitro Study on the Effect of Amorphous Calcium Phosphate and Fluoride Solutions on Color Improvement of White Spot Lesions

**DOI:** 10.3390/dj6030024

**Published:** 2018-06-22

**Authors:** Fahimeh Farzanegan, Hamideh Ameri, Ilnaz Miri Soleiman, Elham Khodaverdi, Abdolrasoul Rangrazi

**Affiliations:** 1 Oral & Maxillofacial Diseases Research Center, Mashhad University of Medical Sciences, Mashhad, Iran; farzaneganf@mums.ac.ir; 2 School of Dentistry, Mashhad University of Medical Sciences, Mashhad, Iran; dr_hamedeh@yahoo.com (H.A.); ilnaz.miri@hotmail.com (I.M.S.); 3 Targeted Drug Delivery Research Center, Pharmaceutical Technology Institute, Mashhad University of Medical Sciences, Mashhad, Iran; khodaverdie@mums.ac.ir; 4 Dental Research Center, Mashhad University of Medical Sciences, Mashhad, Iran

**Keywords:** white spot lesions, ACP, fluoride, colorimeter

## Abstract

The ability of remineralizing agents to improve the color of white spot lesions (WSL) is an important aspect that should be investigated. The aim of this study was to evaluate the effects of 0.05% amorphous calcium phosphate (ACP), 0.5% ACP, and 0.05% fluoride solutions, as well as artificial saliva on the color improvement of white spot lesions (WSLs). In this in vitro study, 50 human premolar teeth were randomly classified into five groups. At baseline, all the samples were assessed by using a colorimeter (E0). Then, white spot lesions were induced on the surface of the teeth by means of a pH-cycling model, and the colorimeter was used again (E1). Afterwards, samples of the 1st and 2nd groups were kept in 0.05% ACP and 0.5% ACP solutions for 1 min/day, respectively. The 3rd group specimens were placed in 0.05% fluoride solution for 1 min/day. The other two groups were kept in artificial saliva and distilled in water separately. All the samples were assessed by the colorimeter for a third time (E2). We found no significant difference between the groups in ∆E1. There was also no significant difference among 0.05% ACP solution, 0.5% ACP solution, 0.05% fluoride solution, and artificial saliva considering ∆E2. However, a significant difference was noted between the above-mentioned solutions and distilled water in ∆E2. With respect to ∆E3, there were considerable differences between ACP solution and artificial saliva. The same results were obtained for the difference between fluoride solution and artificial saliva. However, no significant difference was found among 0.05% ACP, 0.5% ACP, and 0.05% fluoride solutions in terms of ∆E3. In Conclusion, ACP is as effective as fluoride in the color improvement of WSLs and the recommended treatment for this purpose is daily use of 0.05% ACP, 0.5% ACP or 0.05% fluoride solutions.

## 1. Introduction

One prevalent side effect of orthodontic treatments is incipient caries observed as white spot lesions (WSLs) around orthodontic appliances [1,2,3,4,5]. In the demineralization process, enamel loses its color and optical characteristics and takes a white, chalky, opaque appearance which is called WSL. Orthodontic patients are at a higher risk of enamel demineralization. It is difficult for patients to achieve proper oral hygiene in the presences of orthodontic appliances. In addition, due to the existence of bands, brackets, elastics, hooks, and springs, removing dental plaques is a challenging task [6], leading to the formation of WSLs and caries in progressive stages, particularly in those with unacceptable oral hygiene at the pretreatment examination and during treatment [7]. Therefore, it seems essential to adopt some preventive and therapeutic measures before and during orthodontic treatment to lower the probability of developing incipient caries.

It has been revealed that topical fluoride application diminishes the incidence and severity of initial lesions [8,9]. Fluoride can decrease the solubility of tooth minerals by exchanging hydroxyl groups and reducing carbonate content. It can help with mineral precipitation or re-precipitation by decreasing the solubility products of precipitating calcium phosphate [10]. It must be considered that fluoride plays a positive role in the reduction of WSLs and caries. However, overexposure to fluoride leads to adverse effects such as fluorosis. Considering this deficiency of fluoride, an alternative material has been suggested. It has been indicated that amorphous calcium phosphate (ACP) and casein phosphopeptide-amorphous calcium phosphate (CPP-ACP) can cause remineralization of enamel lesions [11]. ACP can suppress demineralization and enhance remineralization by enhancing the buffering effect of saliva [12]. ACP sealant can also increase the level of calcium and phosphate ions via its solubility and induce the formation of apatite [13]. ACP is widely used in dentistry due to its excellent biological and remineralizing properties. Although ACP is mostly used in cream form, we purported to apply it in the form of mouthwash to facilitate its application. Therefore, we produced 0.5% and 0.05% ACP solutions. The remineralizing properties of these materials have been investigated in several studies but one of the main aspects that should be evaluated for the remineralizing agents is their ability to improve the color of the WSLs.

The main objective of this study was to compare the effects of 0.05% ACP, 0.5% ACP, and 0.05% fluoride solutions, as well as artificial saliva on color improvement of WSLs to determine the esthetic enhancement ability of these remineralizing agents.

## 2. Materials and Methods

The samples consisted of 50 human premolars extracted for orthodontic reasons. The inclusion criterion was the absence of any caries, fillings, fractures, or hypoplasia on buccal or lingual surfaces. This study was approved by the Ethics Committee of Mashhad University of Medical Sciences, Mashhad, Iran (ethical code: IR.MUMS.REC.1388.110). At first, the teeth were randomly assigned to five groups. To disinfect, all the samples were placed in 0.1% thymol solution for one week. Following that, samples in each group were stored in 10 mL physiological solution until the outset of the trial. The solution was changed periodically to avoid deterioration.

Lingual, proximal, and root surfaces of the specimens were painted with nail varnish in two layers. Apical foramens were sealed with glue wax. A fiducial hole, with an approximate diameter of 1 mm, was drilled with a slow-speed handpiece at the center of the buccal surface of each tooth. These marks ensured that the same area of the enamel surface was evaluated by the colorimeter. At baseline, buccal surfaces were dried with an absorbent paper. Then, the color of the teeth were determined by using a colorimeter (Shade eye, Shofu, Ratingen, Germany), CIE Lab values were then recorded. The L-value represents the degree of lightness in the Munsell system, a-values demonstrate the position on the red or green (+a = red, −a = green) axes, and b-values reveal the position on the yellow or blue (+b = yellow, −b = blue) axes. All the measurements were carried out by a single operator. The baseline color (E0) was recorded for each tooth and group separately.

At the next stage, WSLs were created on the buccal surface by means of pH-cycling model according to the model of Vieira et al. [14]. Briefly, this stage lasted for 7 days. On the first 5 days, all the samples were stored in demineralization solution (2.0 mmol/L Ca(NO_3_)_2_·4H_2_O, 2.0 mmol/L Na_2_HPO_4_·2H_2_O, 75 mmol/L acetate buffer, 0.04 ppm F, pH 4.7) for 6 h and then in remineralization solution (1.5 mmol/L Ca(NO_3_)_2_·4H_2_O, 0.9 mmol/L Na_2_HPO_4_·2H_2_O, 150 mmol/L KCl, 0.02 mol/L Tris buffer, 0.05 ppm F, pH 7.0) for the next 18 h. On the last 2 days, the specimens were immersed only in remineralization solution. Over this period, the samples were incubated under 37 °C. At the end of this stage, colorimeter was re-used and E1 was determined.

The next stage was the determination of the effects of the mentioned topical medicaments on the buccal surface of the teeth. ACP was prepared by dissolving polyethylene glycol (PEG) in a solution of 0.1 M Ca(NO_3_)_2_. An aqueous solution of (NH_4_)_2_HPO_4_ was added slowly to Ca(NO_3_)_2_ solution and PEG to reach the final ratio of Ca/P = 1.67. During precipitation, pH was adjusted to 10 using NH_4_OH. The reaction was continued for 30 min. Then, the precipitates were washed and freeze-dried to obtain ACP powder. Samples of the 1st and 2nd groups were kept in 0.05% ACP and 0.5% ACP solutions for 1 min/day, respectively. The solutions were adjusted to a pH of 8.5. The 3rd group specimens were put in 0.05% fluoride solution for 1 min/day. The last two groups were assessed as control groups. Samples of the 4th group were put in artificial saliva (Fusayama/Meyer) containing KCl (0.4 g/L), NaCl (0.4 g/L), CaCl_2_·2H_2_O (0.906 g/L), NaH_2_PO_4_·2H_2_O (0.690 g/L), Na_2_S·9H_2_O (0.005 g/L), Urea (1 g/L), and distilled water. Finally, the last group specimens were put in distilled water. This stage lasted 10 weeks. During this period, solutions of the 4th and 5th groups were renewed at 24-hour intervals. For the other three groups, the procedure was different, such that the specimens in each group were immersed in the mentioned solutions and vibrated for 1 min/day. The act of vibrating was the same as the act of tongue, lips, and buccal muscles during the process of mouth rinsing. After that, 5 mL of each solution was replaced with 5 mL of artificial saliva. In this way, we had 5 mL of artificial saliva and 5 mL of the mentioned solutions in each plate. After 30 min of incubation at 37 °C, the samples were cleaned with water and dried with an absorbent paper. For the rest of the day, all the samples of the first three groups were stored in 10 mL of artificial saliva. At the end of 10 weeks, the teeth were assessed by using a colorimeter for a third time (E2). Also, ΔE was defined for each group separately.

Normality of the data distribution and the homogeneity of variances were examined using the Kolmogorov–Smirnov and Levene tests, respectively. To analyze the data, one-way analysis of variance (ANOVA) and Tukey’s test were used at a significance level of *p* < 0.05.

## 3. Results

In this study, color of the teeth was determined in three stages as follows:

1. The initial stage (baseline; E0)
2. The second stage (white spot; E1)
3. The third stage (treatment; E2)

In each condition, L* and *b parameters were measured and color variations were determined according to this formula:

∆E2 = ∆a2 + ∆b2 + ∆L2

In this way, for each group, three ∆E were determined as: ∆E1: color difference between the 1st and 2nd stages, ∆E2: color difference between the 2nd and 3rd stages, and ∆E3: color difference between the 1st and 3rd stages. Descriptive statistics of the ∆E1, ∆E2, and ∆E3 for the five groups are presented in Table 1.

The normality of the data distribution was confirmed by the Kolmogorov-Smirnov test and the homogeneity of variances with the Levene’s test (*p* > 0.05). The results of one-way ANOVA are exhibited in Table 2. None of the groups showed significant differences in ∆E1 (*p* > 0.05). Nonetheless, there were significant differences among the groups with respect to ∆E2 and ∆E3 (*p* < 0.05). Considering ∆E2, no significant difference was found among 0.05% ACP solution, 0.5% ACP solution, 0.05% fluoride solution, and artificial saliva (*p* > 0.05). However, there was a significant difference between the mentioned solutions and distilled water (Group 5) in ∆E2 (*p* < 0.05). According to ∆E3, there were significant differences between the ACP solutions and artificial saliva (*p* < 0.05). The same result was found for the difference between fluoride and artificial saliva (*p* < 0.05). Finally, no significant difference was determined among 0.05% ACP, 0.5% ACP, and 0.05% fluoride solutions considering ∆E3 (*p* > 0.05).

## 4. Discussion

The results of previous studies showed that the incidence of WSLs in orthodontic patients is 50–96% [9]. These lesions develop fast and are largely clinically discovered when they reach a progressive phase. By this time, these lesions are not expected to be repaired naturally. Chapman et al. [7] reported that WSLs mostly involve the facial surfaces of lateral incisors, premolars, canines, and central incisors, respectively. The application of the remineralizing agent is an important strategy against the WSLs. In this way, it is necessary to evaluate the possible esthetic changes in WSLs due to remineralizing agents.

In the present study, fluoride and ACP solutions were used to investigate their ability to improve the color of WSLs, and their effects were compared with artificial saliva and distilled water.

Our study reflected no significant differences among 0.5% ACP solution, 0.05% ACP solution, fluoride solution, and artificial saliva considering ∆E2. To our knowledge, there have been few studies conducted on the color improvement of WSLs by using fluoride or ACP. Most of the studies evaluated the remineralizing properties of these materials. Yuan et al. [15] found no significant differences between NaF and CPP-ACP in esthetic improvements of white-spot lesions. They used 500 ppm NaF solution and CPP-ACP cream for their study. Zachrisson et al. [16] showed that saliva itself can cause regression of WSLs.

Our results showed statistically significant differences in ∆E3 between the ACP groups and the saliva group. Tolcachir et al. [17] found that CPP-ACP penetrated to the depth of the WSL and turned its appearance similar to that of the sound enamel. In their study, CPP-ACP caused a noticeable change in color of WSLs. However, it did not completely improve the esthetic. Heravi et al. [18] evaluated the effectiveness of two creams containing CPP-ACP (MI Paste Plus) and hydroxyapatite and fluoride (Remin Pro) on color improvement of WSLs. Their results showed that both creams have significant reduction in the color difference between sound and demineralized parts of enamel. In other research, Bailey [11] showed that considerably more post-orthodontic WSLs reverted with a remineralizing cream containing CPP-ACP compared with a placebo over 12 weeks. Munjal et al. [19] found that using CPP-ACP for at least 12 weeks is effective for the treatment of WSLs in fixed orthodontic therapy. Concerning ∆E3, the difference between 0.05% fluoride and saliva reached statistical significance. In addition, ∆E3 values were similar in the fluoride and ACP groups. This explains the great efficacy of low-concentrated fluoride (0.05%) and ACP in the treatment of WSLs compared to saliva. Kim et al. [20] found that NaF solution induced both mineral gains and esthetic enhancement of WSLs. Yetkiner et al. [21] observed that low-concentration fluoride treatment improves the color of WSL to some extent. They created artificial WSLs in bovine enamel. In other studies, Kalha [22], Benson et al. [23], and Geiger et al. [24] showed that occurrence of WSLs around orthodontic brackets can be noticeably decreased by the daily use of a 0.05% fluoride mouth rinse. According to the study by Linton [25], 50 ppm of fluoride solution was more useful than 225 ppm of fluoride solution for the remineralization of WSLs. However, Willmot [26] did not detect any benefits in the use of a low fluoride formulation versus a non-fluoride mouth rinse under clinical conditions. On the other hand, Ogaard et al. [27] concluded that high-concentration fluoride solutions are far more effective than low-concentration ones. Bock et al. [28] studied WSL changes in response to weekly 1.25% fluoride gel application after fixed orthodontic treatment. Their results did not show any significant positive effects for high-dose fluoride on post-orthodontic WSL development. He et al. [29] found that the treatment potential of CPP-ACP was not superior to that of fluoride during orthodontic treatment with fixed appliance. On the other hand, in the study by Anderson [30], the number of treated lesions with calcium phosphate was greater than that in fluoride group during a 12-month period.

The major limitation of our study is that the study was performed as an in vitro evaluation and extracted teeth were used, which make it difficult to fully simulate the complex oral conditions.

## 5. Conclusions

In Conclusion, ACP is as effective as fluoride in the color improvement of WSLs and the recommended treatment for this purpose is the daily use of 0.05% ACP, 0.5% ACP or 0.05% fluoride solutions.

## Figures and Tables

**Table 1:** ∆E1, ∆E2 and ∆E3 for five groups.

Groups	∆E1	∆E2	∆E3
0.05% ACP	2.19 ± 0.68	2.29 ± 0.97	1.61 ± 0.86
0.5% ACP	2.13 ± 0.75	1.92 ± 0.41	2.11 ± 0.85
Fluoride	2.30 ± 0.51	2.31 ± 0.66	1.37 ± 0.65
Artificial saliva	2.48 ± 1.01	1.95 ± 1.08	2.88 ± 1.54
Distilled water	2.27 ± 0.84	1.15 ± 0.61	2.51 ± 0.56

**Table 2:** Results of one-way (Analysis of Variance) ANOVA test.

		Sum of Squares	df	Mean Square	F	P
**∆E1**	Between Groups	0.707	4	0.177	0.294	0.88
	Within Groups	27.086	45	0.602	-	-
	Total	27.793	49	-	-	-
**∆E2**	Between Groups	8.852	4	2.213	3.576	0.013
	Within Groups	27.846	45	0.619	-	-
	Total	36.698	49	-		-
**∆E3**	Between Groups	15.487	4	3.872	4.236	0.005
	Within Groups	41.132	45	0.914	-	-
	Total	56.119	49	-	-	-
